# Serologic titers to *Leptospira* in vaccinated pigs and interpretation for surveillance

**DOI:** 10.1371/journal.pone.0260052

**Published:** 2021-11-16

**Authors:** Susan K. Schommer, Nicholas Harrison, Michael Linville, Melissa S. Samuel, Sabrina L. Hammond, Kevin D. Wells, Randall S. Prather

**Affiliations:** 1 National Swine Resource and Research Center, Division of Animal Sciences, University of Missouri, Columbia, Missouri, United States of America; 2 Office of Animal Resources, University of Missouri, Columbia, Missouri, United States of America; UFPL, BRAZIL

## Abstract

Diagnosis and surveillance of pathogenic *Leptospira* is difficult as organisms may be intermittently shed and in small numbers. Therefore, serologic testing by the microscopic agglutination test (MAT) is the primary screening method for leptospirosis. While a MAT titer ≥1:100 is considered to be a positive result, interpretation is complicated by the use of commercial vaccines in pigs. Most guidelines for interpretation of MAT titers in pigs were published in the 1970’s and 1980’s, prior to the development of the current multivalent vaccines. We evaluated MAT titers in routinely vaccinated healthy research pigs compared to their unvaccinated cohorts. Our study confirmed previous reports that the Pomona serovar elicits minimal antibody response even after a second booster 6 months after initial vaccination. However, MAT titers of ≥1:3,200 were detected as early as 4 weeks post initial vaccination for serovars Bratislava and Icterohaemorrhagiae and remained as high as ≥1:1,600 prior to booster at 24 weeks post vaccination. Our study determined that high levels of MAT titers can occur from vaccination alone and high titers are not necessarily indicative of infection. Therefore, the interpretation of MAT titers as indicators of *Leptospira* infection should be readdressed.

## Introduction

Leptospirosis is a global zoonotic disease with a significant impact on both livestock and public health that can be difficult to diagnose. The primary clinical signs in swine are a fever and reproductive problems, which may go undetected depending on their severity and herd management. Leptospirosis can be costly for producers due to decreased reproductive efficiency as well as being a source of infection for on-farm and abattoir workers. Human infection can lead to severe disease and is characterized as flu-like with fatigue, joint pain and, in later stages, kidney damage.

Classification of *Leptospira* was initially based on phenotypic properties of *Leptospira interrogans* which are pathogenic and *Leptospira biflexa* which are saprophytic. Serovars, determined by cross agglutination tests are the basic classification unit of *Leptospira*. Microscopic agglutination testing (MAT) utilizes live organisms as antigen to identify the presumptive serovar in an infection. Based on DNA hybridization there are 25 serogroups of *Leptospira* and over 300 serovars, although only a subset are pathogenic [1]. More recent studies have shown that the correlation between genotypic and serologic classification is poor. This poor correlation may result from the transfer of genes determining serotype between species. Current polymerase chain reaction (PCR) identification methods only identify the *Leptospira* species level, limiting its usefulness for epidemiological studies [2].

Infected swine can result in major economic losses due to abortions, stillbirths, and weak piglets; therefore, vaccination at 3–6 months of age with a bacterin combination of 6 *Leptospira* serovars is common in the United States. *Leptospira interrogans* serovar Bratislava has been isolated from swine herds experiencing reproductive failure since 1991 in the United States and is the most commonly found *Leptospira* serovar in domestic swine; however, because it is endemic regionally pigs infected with that serovar may not show clinical signs [3]. A survey of feral swine in the United States found the Pomona serovar to be the most prevalent with Bratislava closely behind [4]. In some cases, animals infected as fetuses *in utero* and animals that have recovered may become asymptomatic carriers [5, 6]. Carrier animals and individual animals in an endemically infected herd may have MAT titers at or below 1:100 thus further complicating diagnosis and MAT interpretation [7].

Bacterial culture of *Leptospira* is very slow and typically not useful as a principal diagnostic tool. PCR on blood or urine samples can be used to detect acute infections, but the bacteria is cleared from the blood after a few weeks [8] and *Leptospira* organisms may be intermittently shed and then in small numbers [6, 9]. Therefore, PCR is valuable as a diagnostic tool to rapidly detect individual acute infections so that treatment can be initiated and to identify carrier or shedding animals, but many false negatives may occur [10]. Leptospirosis can frequently be subclinical in production animals, consequently the gold standard and primary screening method has remained MAT, particularly at the herd level. MAT is used diagnostically in herds when investigating reproductive loses since those losses are generally observed later in infection when *Leptospira* organisms may be difficult to detect.

Health monitoring and surveillance of herds utilize serological tests because they are cost effective and provide evidence of exposure rather than depending on detection of the pathogen itself. Interpretation of serological results is further complicated if vaccines are used in the herd. Previous studies state that MAT titers are very low from vaccination and are much higher in naturally infected pigs [11–13]. It has been reported that agglutination is not measurable in cattle or pig serum more than 2 months following vaccination, making it an excellent test to detect leptospiral infections [14]. The Leptospirosis Committee of the U.S. Animal Health Association determined in 1977 that the MAT test would be the reference test to improve uniformity of test results. In their description of test interpretation, it states that “MAT titers of 1:100 or greater against one or more serovars are generally considered significant. Diagnosis of leptospirosis may be justified when most of the seropositive animals have titers of 1:1,000 or greater on a single sample” [15]. These statements are decades old and based on previous *Leptospira* vaccines that contained only a single serovar yet are still used for result interpretation today. Current *Leptospira* vaccines usually contain 6 serovars and may be combined with newer adjuvants. Our study reported below was conducted to determine if this interpretation of MAT titers was still true in pigs when using the newer 6 *Leptospira* serovar vaccines with the administration of the recommended booster.

## Materials and methods

### Study protocol

Domestic crossbred pigs 5 to 6 months of age that had not received their first Leptospirosis vaccination were selected for this study from our closed research herd. The 17 eligible pigs representing 5 litters were divided into two groups, litters were evenly distributed between the groups; one group containing 8 pigs remained unvaccinated and another group of 9 pigs received the vaccine. The commercially available Leptospirosis vaccine (FarrowSure Gold B- Zoetis) was given per manufacturer’s instructions including administration of a second dose 4 weeks after the initial vaccination. Animals were co-housed regardless of vaccination status and were in buildings that housed other pigs that were vaccinated according to the recommended protocol (which includes vaccination for Leptospirosis with boosters of breeding aged animals every 6 months unless they are pregnant). Serum was collected for MAT testing at week 0 (pre-vaccination) and at selected time points up to week 34. All pigs received their other vaccinations as per our herd protocol which include immunizations for *Bordetella bronchiseptica*, *Erysipelothrix rhusiopathiae*, *Pasteurella multocida*, Streptococcus, *Mycoplasma hyopneumoniae*, Porcine Circovirus 2, *Haemophilus parasuis*, and *Lawsonia intracellularis*.

### Animals and housing

Pigs used in this study were clinically healthy domestic crossbred animals housed at the University of Missouri-Columbia as part of the National Swine Research and Resource Center herd. Animals were raised on slatted floors with public district water and sewer. Live traps for rodents are utilized and no buildings had evidence of rodents present. Animals were housed socially, fed daily, and had unlimited access to water. Pregnant animals in the vaccination group were excluded from the study prior to the booster as they could not be vaccinated. All animals remained in the research herd at the conclusion of the study. All procedures performed in studies involving animals were approved and conducted in accordance with the ethical standards of the University of Missouri Institutional Animal Care and Use Committee at the University of Missouri in Columbia, Missouri.

### Vaccine

The commercial vaccine used contained inactivated Porcine Parvovirus, Erysipelothrix rhusiopathiae, and *Leptospira* serovars Canicola, Grippotyphosa, Hardjo, Bratislava, Icterohaemorrhagiae and Pomona. The vaccine was administered intramuscularly.

### Samples

Serum was collected in heparinized tubes by jugular venipuncture at day 0 prior to vaccination, prior to booster 4 weeks post vaccination (wpv), and 6 weeks post-booster (10 wpv). At 24 wpv a 2^nd^ booster was given to any non-pregnant animals, serum was again collected prior to the booster then 4 weeks (28 wpv) and 10 weeks post-vaccination booster (34 wpv). Pregnant animals in the vaccination group were excluded after sampling 24 wpv, since they could not receive the booster. Serum sampling tubes were centrifuged in a tabletop centrifuge (Eppendorf 5810R) for 10 minutes at 3,000 g. Serum was separated from the clot and refrigerated until shipment. Whole blood was collected in a similar manner by using EDTA tubes. Urine was collected by using free catch. Whole blood and urine sample collection was done at 10 and 24 wpv.

### MAT testing

Microscopic agglutination test (MAT) testing requires live cultures and expertise in the technique; therefore, serum was submitted to Iowa State University, a fully accredited lab, for the 6-way *Leptospira* MAT, which uses *L*. *interrogans* Bratislava, Canicola, Grippotyphosa, Hardjo, Icterohaemorrhagiae, and Pomona serovars. The initial MAT titer dilution performed is 1:100 and the final dilution is 1:12,800. Final titer is determined to be the highest dilution where 50% or more of the well shows agglutination.

### PCR testing

PCR testing was performed on blood and centrifuged urine (ABI 7500Fast, Applied Biosystems) by using the Quick DNA Miniprep Plus kit (Zymo Research) for DNA extraction and the QuantiFast Pathogen + IC kit (Qiagen) by using published protocols [16]. The protocol was modified to use the internal control primers provided with the PCR reagents as a single tube reaction and optimized by using serial dilutions of extracted *Leptospira* cultures. Briefly, 4 μl of high concentration internal control was added to the sample prior to extraction. The PCR reaction mix contained 0.4 μM of the forward and reverse lipL primers [16], 1.3 μM lipL probe, and 1X of the master mix components (Buffer, 50X Rox, Internal control primers) plus water and 5 μl of extracted sample for a total reaction volume of 25 μl. PCR was performed at 95°C for 5 minutes followed by 45 cycles of 95°C for 15 seconds and 60°C for 30 seconds. All Urine samples were also tested by PCR at the Iowa State University Diagnostic Laboratory.

## Results

**Fig 1:**
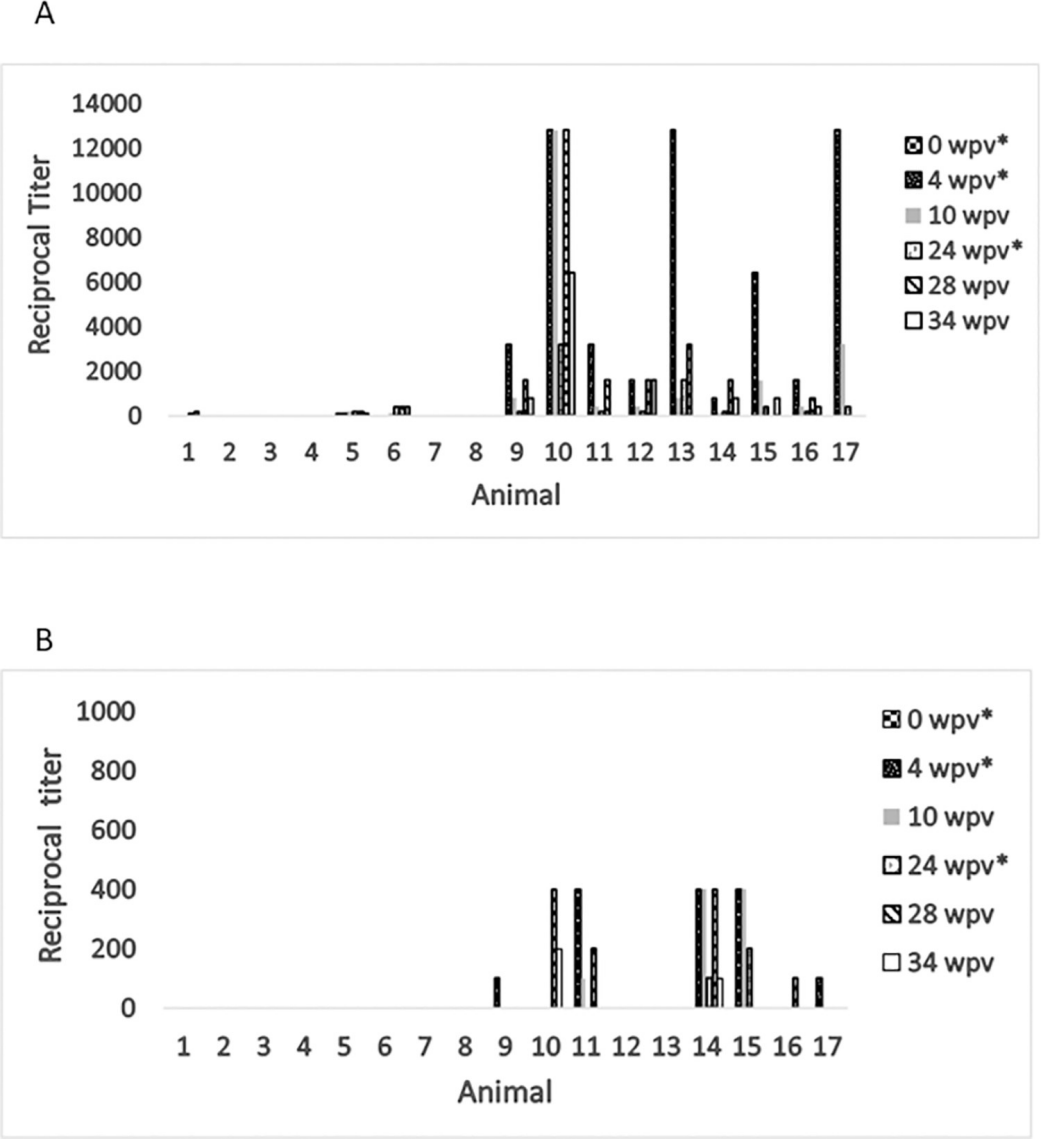
MAT reciprocal titers of *Leptospira* in unvaccinated and vaccinated animals vary by animal and serovar. Serum from each animal was assayed by MAT for Bratislava (A) and Pomona (B) for *Leptospira* agglutination. Blood was collected prior to vaccination on days vaccine was administered (*). Pigs 1–8 did not receive the vaccine at any time, pigs 9–17 received the vaccine.

Serum samples were collected from 17 pigs at 5 to 6 months of age, prior to their first *Leptospira* vaccination, and MAT testing was performed. The day 0 timepoint had no pigs with a titer greater than 1:100 (Table 1). While a few pigs in the unvaccinated group did have low level titers to some serovars, at no time in the study did any unvaccinated animal have a titer greater than 1:800 for any serovar (Table 2). In contrast to the results of the unvaccinated animals, 9 out of 9 vaccinated pigs were positive for serovars Icterohaemorrhagiae and Bratislava at multiple timepoints (Table 1), with all but 1 of those also testing at higher than 1:1,000 at some time point (Table 3 and Fig 1A). However, for the other serovars the majority of vaccinated animals never reached a titer greater than 1:100, and 1:400 was the maximum titer in any animal for serovars Pomona, Canicola, Hardjo, and Grippotyphosa.

**Table 1:** Percentage of pigs with a reciprocal MAT titer >100 for each serovar.

	Unvaccinated control						Vaccinated Cohorts					
	(Time point wpv*)						(Time point wpv*)					
Serovar	0	4	10	24	28	34	0	4	10	24	28	34
Pomona	0	0	0	0	0	0	0	33	22	11	43	33
Icterohemorrhagiae	0	13	13	13	13	13	0	100	100	78	100	100
Canicola	0	0	13	13	13	13	0	33	22	0	29	0
Hardjo	0	0	0	0	0	0	0	33	0	11	0	0
Grippotyphosa	0	0	0	0	0	0	0	33	11	0	43	0
Bratislava	0	0	13	25	38	0	0	100	89	100	100	100

*wpv = weeks post vaccination

**Table 2:** MAT titers in unvaccinated pigs, only serotypes that had a positive result are included.

Animal ID and serotype	Weeks post study initiation					
	0	4	10	24	28	34
1 Bratislava	0	0	0	100	200	nd
4 Icterohemorrhagiae	100	200	400	800	400	800
5 Canicola	0	0	400	200	400	400
5 Bratislava	100	100	200	200	200	100
6 Bratislava	0	0	100	400	400	400

**Table 3:** Individual animal MAT titers for *Leptospira* Icterohemorrhagiae across 34 weeks post initial vaccination.

	Reciprocal MAT titer at weeks post vaccination					
Unvaccinated Animal	0	4	10	24	28	34
1	0	0	0	0	0	nd
2	0	0	0	0	0	0
3	0	0	0	0	0	0
4	100	200	400	800	400	800
5	0	0	0	0	0	0
6	0	0	0	0	0	0
7	0	0	0	0	0	0
8	0	0	0	0	0	0
Vaccinated Animal						
9	0	6400	400	100	3200	1600
10	0	6400	6400	1600	≥12800	6400
11	0	6400	6400	200	800	nd
12	0	6400	400	100	800	1600
13	0	1600	800	200	800	nd
14	0	1600	800	400	1600	800
15	0	≥12800	1600	800	nd	800
16	0	1600	800	200	400	200
17	0	6400	1600	400	0*	nd

*Bled at the time of booster, post farrowing 35wpv.

nd = not done.

Two unvaccinated animals did consistently have low level titers to some serovars. Animal 5 had titers of 1:200 and 1:400 for the Canicola serovar starting at 10 weeks into the study and continuing throughout and animal 4 had Icterohaemorrhagiae titers ranging from 1:100 to 1:800 at every study timepoint (Table 2). Bratislava titers were the most commonly found in unvaccinated animals with three pigs having titers at weeks 24 and 28, though most titers were 1:200 or less (Table 2). *Leptospira* PCR testing was added to the study to further investigate the source of the MAT titers in unvaccinated animals. Urine collected from pig 4 at the 24 and 28 weeks post vaccination (wpv) time point and submitted to the Iowa State VDL for *L*. *interrogans* PCR testing was found to be negative. Blood samples from pigs in this study were also tested in-house for *Leptospira* by PCR and all tested negative (S1 Table). In addition, no animals in the study exhibited any clinical signs of leptospirosis, and PCR on blood and urine from surveillance testing of other animals in the herd was negative prior to and during the course of the study.

Fig 1A illustrates the variability in response to serovar Bratislava after vaccination. Most animals reached their peak titer at 4 weeks after their initial vaccination, having a lower response after their second booster at 24 wpv except for animal 10. Three out of 9 pigs were positive at the maximal dilution of 1:12,800, yet 5 animals never had titers greater than 1:3,200. The unvaccinated group had one animal (pig 5) with a titer of 1:400 for the last 3 timepoints, while 5 unvaccinated pigs never exhibited any titer.

Vaccinated pigs reacted similarly to serovar Icterohaemorrhagiae. Most animals reached their peak titer at 4 weeks after their initial vaccination, and animal 10 was the most reactive (Table 3). Pig 4 was the only unvaccinated animal to react to serovar Icterohaemorrhagiae at any timepoint.

The Pomona serovar has been consistently noted to elicit a minimal antibody response in vaccinated animals [13, 17]. Unvaccinated animals did not have any positive MAT titers to *L*. *interrogans* Pomona during the course of this study and even vaccinated animals never had a MAT titer greater than 1:400 (Fig 1B).

## Discussion

Monitoring herds for *Leptospira* infection is important for both the swine and the human caretakers. Even with proper vaccination of swine, while minimizing clinical signs, infection and shedding can still occur and a human risk still exists [17]. Recognizing clinical symptoms of leptospirosis can be a challenge and in livestock diagnosis of leptospiral infection typically relies on serology. Demonstrating high herd health or negative status for animal movement also primarily uses MAT testing. Interpretation of those serologic results is not always clear cut especially when vaccination is being used.

MAT results at a 1:100 dilution are considered to be a positive result, although not necessarily a level indicative of infection. Conversely, herds endemically infected with pig-adapted strains of Bratislava, may have very low MAT titers of under 1:100 [18]. Much of the published literature on MAT titers in pigs is from the 1970s and 1980s when vaccination was less common and those vaccines contained only one or two serovars [14, 17]. Today’s vaccines typically contain 6 serovars and are commonly combined into a reproductive vaccine with Erysipelas and Porcine Parvovirus. More recent publications have suggested that titers ≥1:1,000 are highly suggestive of leptospirosis in swine when showing recent clinical signs [19] and when performing a retrospective diagnosis if the majority of the animals in the tested herd have titers over 1:1,000 [20].

In our study we have demonstrated that swine can develop MAT titers to serovars Bratislava and Icterohaemorrhagiae at levels of 1:3,200 and above after routine vaccination. Because the titers of only a few serotypes are elevated in the vaccinated animals these results can easily be confused with natural infection, specifically Bratislava as it is the serovar of primary concern in US swine [3, 21]. In the current study the six serovars tested for in the MAT assay are the same serovars included in the vaccine formulation administered. It is not known why pigs respond unevenly to the six different serovars. A previous study also demonstrated that vaccination could significantly increase titers to *Leptospira* serovars Canicola, Grippotyphosa, and Icterohaemorrhagiae, but those titers averaged less than 1:400 [12]. The Bratislava serovar was not present in any of the vaccine formulations at that time. Our results indicate that the strongest antibody response to the *Leptospira* vaccine occurs after the first vaccination. The vaccinated group had declining titers six weeks after the second vaccination (10 wpv) that continued to decline through their booster at 24 wpv. Only two pigs, 10 and 14, matched or exceeded their initial post-vaccination MAT titer demonstrating that the highest titers were not a result of repeated vaccination.

Minor variations in MAT titers can occur. For example, a single vaccinated animal had a titer for the Bratislava serovar of 1:100 at 10 wpv followed by a 1:200 titer at 24 wpv; the difference of a single dilution. Additionally, for the Hardjo serovar only 4 vaccinated animals showed a titer greater than 1:100, each reached 1:400 at a single timepoint. Both of those instances resulted in a case where an additional animal was positive at 24 wpv that was not positive at 10 wpv (Table 1). Some variation in MAT results is expected based the assay reliance on subjective interpretation. All samples were tested at the same diagnostic laboratory; however, they were not all tested simultaneously which could result in slightly discrepant results due to differences in technicians and bacterial cultures used [22].

Four of the eight unvaccinated animals had low level MAT titers to *Leptospira* at some time during the study. None of the pigs exhibited a fourfold rise in titer in successive timepoints, which is the CDC and World Organisation for Animal Health (OIE) definition of acute infection, and only a single sample from an unvaccinated animal reached a MAT titer of ≥800 (Table 2). As this result was unexpected, PCR for *Leptospira spp*. was performed on urine from Animal 4 and blood from other animals in this study to confirm that there was not circulating *Leptospira*. There were no clinical signs of leptospirosis in the herd; additional animals outside of the study group were also tested and determined negative for *Leptospira* by PCR on blood both during and prior to initiation of the study as part of our health surveillance program. While specificity of MAT testing is relatively high, a previous study on human *Leptospira* cross-reactivity demonstrated that MAT false positives can occur in the presence of other diseases including Cytomegalovirus and Viral hepatitis [23] which both occur frequently in pigs [24, 25]. The use of *Leptospira* PCR on urinary samples in Brazilian livestock demonstrated that serological and molecular results were discrepant regardless of MAT titer and even at MAT titers indicative of active disease, based on OIE interpretation, PCR results were negative in 78% of the animals [10]. This is likely due to intermittent shedding of the organism in livestock [26] but could also be due to PCR inhibitors in urine. Blood samples may avoid some of those problems and have been shown to be equivalent to urine samples in humans and dogs [27, 28]. It is important to note that no other unvaccinated animals in the study had any titer to the Icterohemorrhagiae serovar at any timepoint despite animals being co-housed.

Our study had a relatively low number of age-matched and co-housed animals, yet it demonstrated clearly that high MAT titers could result from vaccination alone. This complicates interpretation of MAT results in vaccinated pigs for both surveillance and diagnostics, yet PCR for detection of *Leptospira* organisms is dependent on shedding and timing of sampling therefore not suitable for surveillance. Further studies on the newer vaccines could elucidate if this is vaccine specific and if the same serovars are the most stimulated by other vaccine formulations. If the same titers are increased by other vaccine formulations, then increased titers in those serovars must also be interpreted differently than had been recommended in the past.

## Supporting information

S1 Table Pathogenic *Leptospira* PCR results. (DOCX)
