# Supplementary material for: Serologic titers to Leptospira in vaccinated pigs and interpretation for surveillance
Source: PLoS One. 2021 Nov 16;16(11):e0260052. doi: 10.1371/journal.pone.0260052 (PMC8594815; doi:10.1371/journal.pone.0260052)
Supplement: S1 Table — (DOCX) [file pone.0260052.s001.docx]

S1 Table. Real-time PCR for Pathogenic *Leptospira* spp

| **Animal ID** | **Sample timepoint (wpv)** | **Sample type** | **Real-time PCR result** |
| --- | --- | --- | --- |
| 1 | 10 | blood | no Ct |
| 1 | 28 | blood | no Ct |
| 2 | 28 | blood | no Ct |
| 3 | 10 | blood | no Ct |
| 3 | 24 | urine | no Ct |
| 3 | 28 | blood | no Ct |
| 4 | 10 | blood | no Ct |
| 4 | 24 | urine | no Ct |
| 4 | 28 | blood | no Ct |
| 4 | 28 | urine | no Ct |
| 5 | 10 | blood | no Ct |
| 5 | 28 | blood | no Ct |
| 6 | 10 | blood | no Ct |
| 6 | 28 | blood | 40.9 (neg cut off 37) |
| 7 | 10 | blood | no Ct |
| 7 | 28 | blood | no Ct |
| 8 | 10 | blood | no Ct |
| 8 | 28 | blood | no Ct |
| 9 | 10 | blood | no Ct |
| 9 | 28 | blood | no Ct |
| 10 | 28 | blood | no Ct |
| 11 | 10 | blood | no Ct |
| 11 | 28 | blood | no Ct |
| 12 | 28 | blood | no Ct |
| 13 | 10 | blood | no Ct |
| 13 | 24 | urine | no Ct |
| 13 | 28 | blood | no Ct |
| 14 | 4 | blood | no Ct |
| 14 | 28 | blood | no Ct |
| 15 | 4 | blood | no Ct |
| 15 | 28 | blood | no Ct |
| 16 | 10 | blood | no Ct |
| 16 | 28 | blood | no Ct |
| 17 | 10 | blood | no Ct |
| 17 | 28 | blood | no Ct |
